# Multilevel Venous Obstruction in Patients with Cardiac Implantable Electronic Devices

**DOI:** 10.3390/medicina60020336

**Published:** 2024-02-17

**Authors:** Marek Czajkowski, Anna Polewczyk, Wojciech Jacheć, Jarosław Kosior, Dorota Nowosielecka, Łukasz Tułecki, Paweł Stefańczyk, Andrzej Kutarski

**Affiliations:** 1 Department of Cardiac Surgery, Medical University of Lublin, 20-059 Lublin, Poland; mczajkowski@interia.pl; 2 Institute of Medical Sciences, Jan Kochanowski University, 25-317 Kielce, Poland; 3 Department of Cardiac Surgery, Świętokrzyskie Center of Cardiology, 25-736 Kielce, Poland; 4 2nd Department of Cardiology, Faculty of Medical Sciences in Zabrze, Medical University of Silesia in Katowice, 41-800 Zabrze, Poland; wjachec@interia.pl; 5 Department of Cardiology, Masovian Specialist Hospital of Radom, 26-617 Radom, Poland; jaroslaw.kosior@icloud.com; 6 Department of Cardiology, The Pope John Paul II Province Hospital of Zamość, 22-400 Zamość, Poland; dornowos@wp.pl (D.N.); paolost@interia.pl (P.S.); 7 Department of Cardiac Surgery, The Pope John Paul II Province Hospital of Zamość, 22-400 Zamość, Poland; luke27@poczta.onet.pl; 8 Department of Cardiology, Medical University of Lublin, 20-059 Lublin, Poland; a_kutarski@yahoo.com

**Keywords:** multilevel lead-related venous obstruction, risk factors, transvenous lead extraction, complications, long-term survival

## Abstract

*Background and Objectives*: The nature of multilevel lead-related venous stenosis/occlusion (MLVSO) and its influence on transvenous lead extraction (TLE) as well as long-term survival remains poorly understood. *Materials and Methods*: A total of 3002 venograms obtained before a TLE were analyzed to identify the risk factors for MLVSO, as well as the procedure effectiveness and long-term survival. *Results*: An older patient age at the first system implantation (OR = 1.015; *p* < 0.001), the number of leads in the heart (OR = 1.556; *p* < 0.001), the placement of the coronary sinus (CS) lead (OR = 1.270; *p* = 0.027), leads on both sides of the chest (OR = 7.203; *p* < 0.001), and a previous device upgrade or downgrade with lead abandonment (OR = 2.298; *p* < 0.001) were the strongest predictors of MLVSO. *Conclusions*: The presence of MLVSO predisposes patients with cardiac implantable electronic devices (CIED) to the development of infectious complications. Patients with multiple narrowed veins are likely to undergo longer and more complex procedures with complications, and the rates of clinical and procedural success are lower in this group. Long-term survival after a TLE is similar in patients with MLVSO and those without venous obstruction. MLVSO probably better depicts the severity of global venous obstruction than the degree of vein narrowing at only one point.

## 1. Introduction

Venous obstruction is a common complication in patients with cardiac implantable electronic devices (CIED) and has been assessed in numerous studies [1,2,3,4,5,6,7,8,9,10,11,12,13,14,15,16,17,18,19,20,21,22,23]. However, our understanding of the severity and risk factors for multilevel lead-related venous stenosis/occlusion (MLVSO) remains limited. Venous stenosis/occlusion is usually asymptomatic, but it may become clinically important in the event of a device replacement or upgrade and the insertion of anesthetic lines and different types of catheters, especially dialysis catheters. In earlier studies, we evaluated the effect of lead-related venous obstruction on the level of difficulty and the associated risk of a transvenous lead extraction (TLE) and analyzed the location and pathophysiology of venous obstruction in CIED patients [24,25,26]. The purpose of this study is to create a “venous obstruction map” in patients with intracardiac leads based on a large database of patients undergoing transvenous lead extraction.

## 2. Methods

### 2.1. Study Population

This post-hoc analysis used clinical data of 3002 patients who underwent a transvenous lead extraction (TLE) between June 2008 and June 2021 by one main operator at three high-volume centers. All information concerning patients and procedures was inserted into the computer database. The patients with medical contraindications for intravenous contrast administration were excluded from the study. An eGFR of 60 mL/min /1.73 m^2^ was a cut-off value, but if the results of venography could impact the procedural strategy, the contrast injection was performed at slightly lower eGFR values.

This report comes from three high-volume centers conducted by one team performing annually more than 200 TLEs.

### 2.2. Venography Procedure

An intravenous catheter for the pre-extraction venography was placed in the peripheral vein in the arm on the side or sides of the lead implantation. All patients received an injection of 20–40 mL high-quality contrast medium (350 mg iodine/mL), and the flow of contrast in the upper arm, neck, and thoracic veins was recorded by cineangiography in the anteroposterior view as previously described [24,25,26].

All available venograms were retrospectively reviewed by an extraction team, consisting of an experienced electrophysiologist and cardiothoracic surgeon with more than 30 years of experience in thoracic vein catheterization for various purposes.

**Figure 1:**
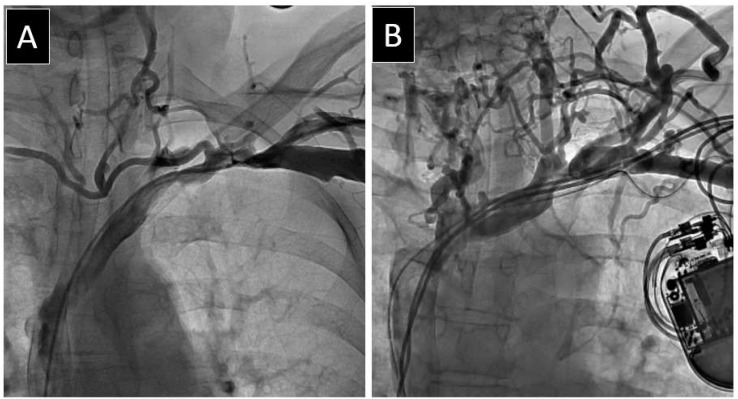

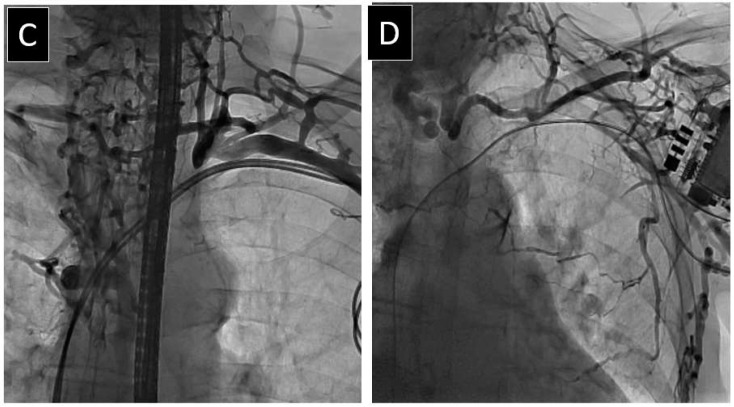
Examples of lead-related obstruction of the major thoracic veins. Leads on the left side of the chest. A transesophageal echocardiographic probe can be seen on several images. (**A**) Severe stenosis isolated to one vein (subclavian). (**B**) Moderate stenosis affecting two veins (subclavian and brachiocephalic). (**C**) Three veins affected. Brachiocephalic vein occlusion and severe stenosis of subclavian and superior vena cava veins. (**D**) Four veins affected. Occlusion of axillary, subclavian, and brachiocephalic veins with severe stenosis of superior vena cava. The extent of collateral circulation through neck (**A**–**D**) and thoracic (**B**,**D**) veins depends on the degree of obstruction.

**Figure 2:**
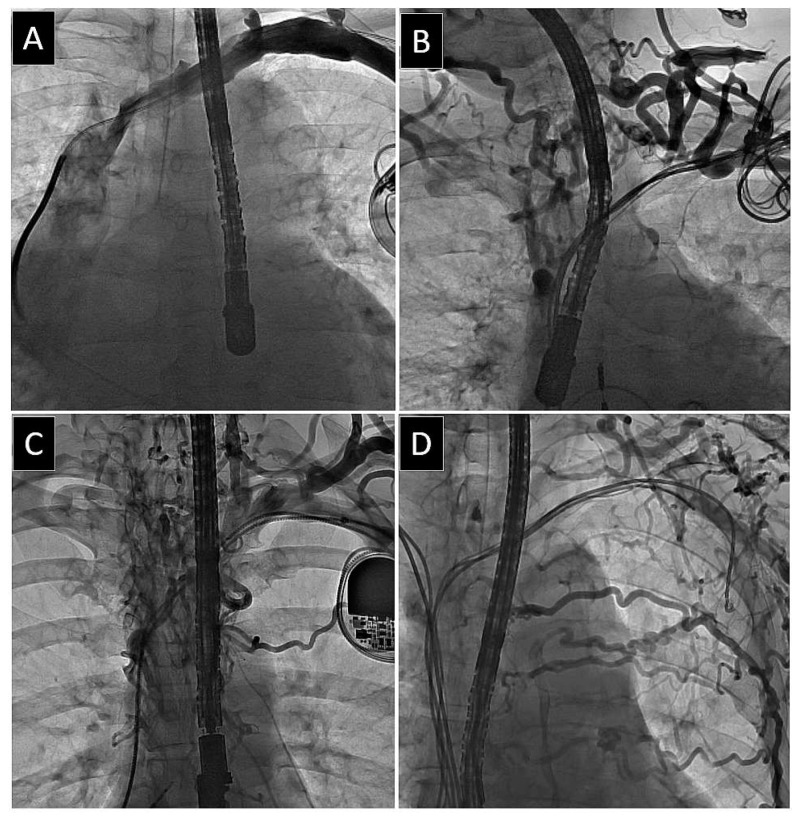
Examples of lead-related obstruction of the major thoracic veins. Leads on the left side of the chest. A transesophageal echocardiographic probe can be seen. (**A**) Severe stenosis isolated to one vein (brachiocephalic). (**B**) Two veins affected. Occlusion of subclavian and brachiocephalic veins. (**C**) Three veins affected. Stenosis of subclavian vein, occlusion of brachiocephalic vein, and severe stenosis of superior vena cava. (**D**) Four veins affected. Occlusion of axillary, subclavian, and brachiocephalic veins, and severe stenosis of superior vena cava. Well-developed collateral circulation through neck and thoracic (**B**–**D**) veins.

**Figure 3:**
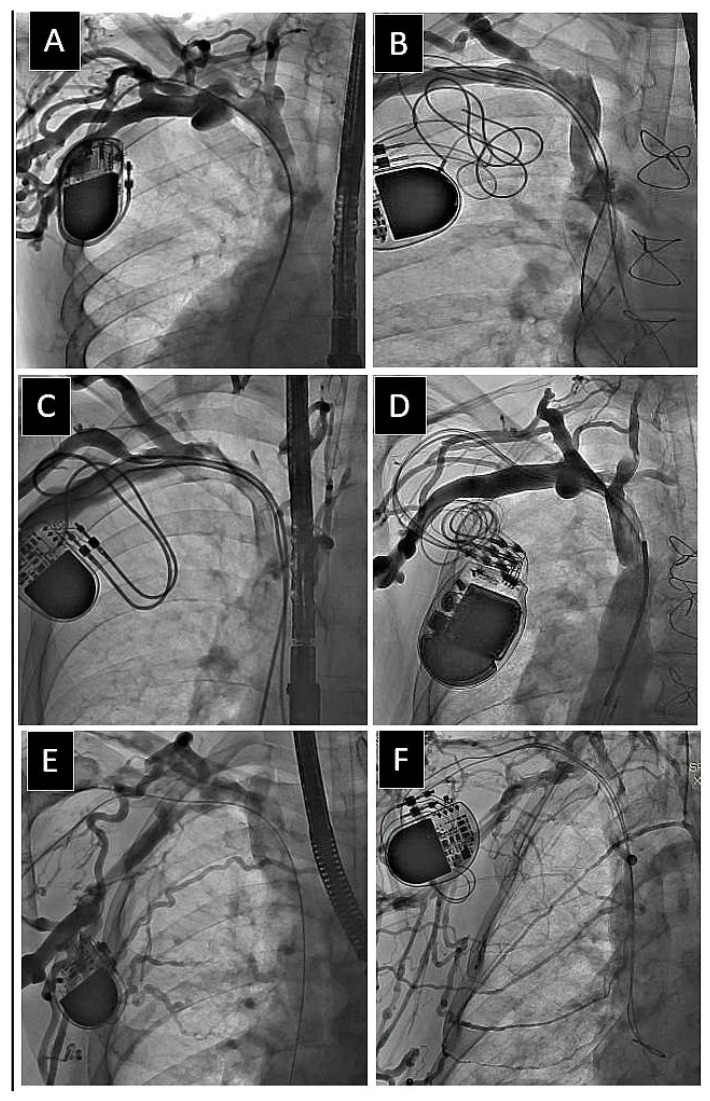
Six examples of lead-related obstruction of the major thoracic veins in patients with leads on the right side of the chest. A transesophageal echocardiographic probe can be seen. (**A**,**B**) Moderate stenosis isolated to one vein (right brachiocephalic vein). (**C**,**D**) Two veins with severe stenosis (right subclavian and right brachiocephalic veins). (**E**,**F**) Three veins affected. Severe stenosis of right subclavian and brachiocephalic veins and severe stenosis of superior vena cava. Collateral circulation through neck (**A**,**D**,**F**) and thoracic (**E**,**F**) veins.

A map of venous patency was created for the axillary vein (AxV), the subclavian vein (SCV), the brachiocephalic (anonymous) vein (AnV), and the superior vena cava (SVC) as previously described [26]. We identified the narrowest (stenotic) and widest (non-stenotic) points to obtain the venous diameter. Additional measurements and drawings were documented as static images. In case of doubt, the blood flow was assessed from video recordings. The final status of the vein was determined in the anteroposterior view as follows: (1) patent, i.e., no stenosis in plain view, (2) mild stenosis (<30% reduction in plain view), (3) moderate stenosis (30–60%), (4) severe stenosis (≥60% in plain view), and (5) complete occlusion (100%) (Figure 1, Figure 2 and Figure 3).

### 2.3. Lead Extraction Procedure

Lead extraction procedures were performed according to the most recent guidelines (HRS 2017 and EHRA 2018) [27,28,29]. The indications for the TLE and the definitions of periprocedural complications were established according to the 2017 HRS Expert Consensus Statement on Cardiovascular Implantable Electronic Device Lead Management and Extraction [28].

As previously described, most procedures were performed [24,25,26] using polypropylene Byrd dilator sheaths (Cook^®^ Medical, Leechburg, PA, USA), mainly via the implantation venous entry site. If technical difficulties arose, a different vascular access and/or additional tools such as Evolution (Cook^®^ Medical, Leechburg, PA, USA), TightRail (Phillips, Cambridge, MA, USA), lassos, or basket catheters were utilized. Excimer laser sheaths were not used by the team.

### 2.4. Approval of the Bioethics Committee

All patients gave their informed written consent to undergo a TLE and for the use of anonymous data from their medical records. The protocol was approved on 18 November 2018 by the Bioethics Committee at the Regional Chamber of Physicians no. 288/2018/KB/VII. This study was carried out in accordance with the ethical standards of the 1964 Declaration of Helsinki.

### 2.5. Statistical Analysis

In this study, the patients were divided into four groups according to the number of affected veins: group 1—no significant narrowing, group 2—one vein with significant narrowing (grades 3, 4, and 5), group 3—two veins with significant narrowing (grades 3, 4, and 5), group 4—three, four, five, or more veins with significant narrowing (grades 3, 4, and 5). For uniformity, all continuous variables are presented as the mean and standard deviation. The categorical variables are presented as numbers and percentages. Due to the different sizes of the study groups, nonparametric tests were used. The Kruskal–Wallis ANOVA test was used first to determine whether the data influenced the multilevel nature of the venous stenosis/occlusion. Then, the variables with a *p*-value < 0.1 were compared using the nonparametric Chi^2^ test with Yates correction (dichotomous data) or the unpaired Mann–Whitney U test (continuous data), as appropriate. 

Comparisons were made between group 1 and groups 2, 3, and 4 together; group 1 and groups 3 and 4 together, and between group 1 and group 4. All variables with a *p*-value less than 0.05 (group 1 vs. groups 2, 3, and 4 together) were included in the multivariable linear regression model. Of all derivative variables (which were highly correlated), only one was included in the multivariate model. This especially applied to the number of leads, abandoned leads, age of leads, and patient’s age. Among these parameters, the patient’s age at the first CIED implantation, the number of leads, the history of the device upgrade or downgrade with lead abandonment, and the cumulative lead dwell time were included in the multivariate analysis.

Additionally, the Spearman r correlation was used to determine the relationship between the MLVSO severity and the number of affected veins, number of serious technical problems, and procedure time.

Kaplan–Meier survival curves were plotted to assess the effect of MLVSO on mortality and compared using the log-rank test. A *p*-value less than 0.05 was considered statistically significant.

The statistical analysis was performed using Statistica 13.3 (TIBCO Software Inc., Palo Alto, CA, USA).

## 3. Results

### 3.1. Study Population

The study population consisted of 3002 patients (with a mean age of 66.93 years, 39.34% females). The most common underlying disease in patients undergoing a TLE was ischemic heart disease (57.56%). The mean left ventricular ejection fraction (LVEF) was 49.04%, and the mean NYHA class was 1.84. The most common comorbidity was arterial hypertension (58.99%). Diabetes and renal failure were observed in 20.35% and 19.75% of patients, respectively. The mean creatinine level was 1.17 mg/dL. Atrial fibrillation was present in 23.05% of patients, and 40.14% of patients required long-term anticoagulation. The Charlson comorbidity index was 4.77 points on average.

### 3.2. Patient Groups

Four groups of patients were selected for analysis of venous obstruction: group 1: no significant narrowing (only grades 1 and 2; 1108 patients); group 2: one vein with significant narrowing (grades 3, 4, and 5; 1152 cases); group 3: two veins with significant narrowing (grades 3, 4, and 5; 665 cases); group 4: three, four, five, or more veins with significant narrowing (grades 3, 4, and 5; 77 patients). The total number of patients was 3002.

Comparative analysis showed that the patient’s age at the first system implantation, normal ventricular diameter, permanent atrial fibrillation, arterial hypertension, congenital or unknown underlying heart disease, body mass index, and Charlson comorbidity index were associated (directly or indirectly) with a larger number of affected veins and the extent of the venous obstruction (Table 1).

A detailed analysis of the procedural factors associated with the development of MLVSO showed a relationship between the severity of venous obstruction defined as the number of significantly affected veins (grades 3 to 5) and the presence of lead-related infective endocarditis (LRIE) with or without pocket infection, the type of device (AAI, VVI, DDD, or CRT-P), the number of leads in the heart (including abandoned leads), the leads located on the left or both sides of the chest, the history of upgrades or an additional lead implantation, and a longer lead dwell time (Table 2).

**Figure 4:**
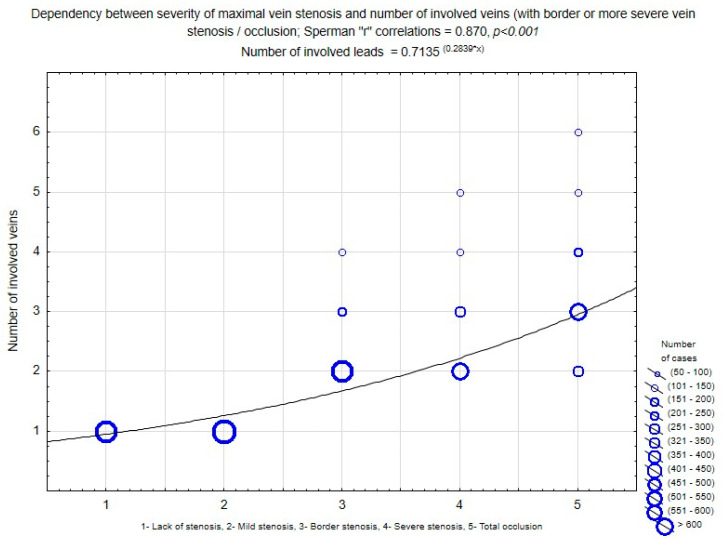
Relationship between the severity of maximal vein stenosis and the number of affected leads (with borderline or more severe venous stenosis/occlusion); Spearman “r” correlations = 0.870. *p* < 0.001, and number of leads = 0.7135^(0.2839 × x)^.

A detailed analysis of the severity of obstruction in different veins showed that the degree of narrowing at the site of maximal stenosis corresponded to the number of significantly affected veins (Spearman r coefficient = 0.870; *p* < 0.001) (Figure 4). (Supporting information see Supplementary File).

### 3.3. Univariable and Multivariable Regression Analysis of Factors Influencing Multilevel Lead-Related Venous Stenosis/Occlusion

In univariable regression analysis (Table 3) there was a positive relationship between MLVSO (minimum two-level venous obstruction) and the patient’s age at the first CIED implantation and during the TLE, the number of leads in the heart (including abandoned leads), the presence of a coronary sinus lead, leads on both sides of the chest, and the age of the leads, which is the cumulative dwell time of the implanted leads. On the other hand, MLVSO influenced the occurrence of infectious complications including LRIE with or without pocket infection. In patients with MLVSO, a system downgrade and removal with deferred reimplantation was more often an indication for the TLE. A multivariable regression analysis revealed that the most important factors predisposing to MLVSO (minimum two-level) were the patient’s age at the first system implantation (OR = 1.015; *p* < 0.001), the number of leads in the heart (OR = 1.556; *p* < 0.001), a CS lead presence (OR = 1.270; *p* = 0.027), leads on both sides of the chest (OR = 7.203; *p* < 0.001), and a previous upgrade or downgrade with lead abandonment (OR = 2.298; *p* < 0.001). Both permanent atrial fibrillation (OR = 0.547; *p* < 0.001) and a higher body mass index (OR = 0.864; *p* < 0.001) seemed to offer some protection against MLVSO (Table 3).

An analysis of the effectiveness and safety of the TLE in the individual study groups showed that any major complication (hemopericardium, hemothorax, or significant tricuspid valve damage during the TLE), minor complications, and partial radiographic success (remained tip or <4 cm lead fragment) were most likely to occur in patients with significant narrowing affecting three, four, or five veins. On the other hand, the chances of complete clinical success and complete procedural success were lower in patients with a higher number of significantly narrowed or occluded veins (grades 3–5). An analysis of survival in patients with MLVSO undergoing a TLE showed no differences in the percentage of deaths over the 3-year follow-up period (Table 4).

## 4. Discussion

In previous studies exploring venous complications in CIED patients, lead-related venous obstruction has been categorized according to the degree of maximal narrowing [1,2,3,4,5,6,7,8,9,10,11,12,13,14,15,16,17,18,19,20,21,22,23]. Mild narrowing was found in 10% [2,7], 23% [19], and 40% [2,7,11]; moderate narrowing in 6–8% [2,11,16], 13–12% [13,19,20], and 23–50% [1,14]; and severe narrowing/total occlusion in 3–9% [2,7,11,13,20] and 11–22% [1,12,14,15,19] of patients. Some earlier studies based on single measurements of the maximal stenosis also provided unclear conclusions [24,25,26]; therefore, we hypothesized that the number of significantly narrowed veins (grades 3–5) would better reflect the foreign body response to the implanted lead. The analysis of more than 3000 venograms in this study suggests that the degree of maximal narrowing at one site is not a measure of the extent of venous scarring. The number of veins with a significant obstruction (grades 3–5) showed a relationship between the occurrence of single maximal narrowing and the number of significantly affected veins (the extent of narrowing). For this reason, the number of affected veins was used as a measure of vessel wall degeneration in the presence of leads.

There have been many reports on the potential risk of lead-related venous obstruction, but the findings are inconsistent because the studies were performed in relatively small cohorts of patients [4,5,6,7,8,9,10,11,12,13,16,17,18,20]. Low left ventricular EF [7,10,19], permanent AF [7,17], and no anticoagulation and antiplatelet treatment [6,20] were identified as risk factors for venous obstruction in CIED patients. So far, however, there have been no investigations that assess the importance of multilevel venous obstruction. This present study, carried out on a population many times larger than the previous ones, demonstrates that the number of leads is the most important factor influencing the development of MLVSO. There is much disagreement about the importance of lead burden as a risk factor for venous obstruction. Some investigators support the view [6,9,13,17,20], others argue against [1,4,5,8,10,15,16,18,21]. The current study shows that 4 and >4, 5 and >5 leads in the heart, leads placed on both sides of the chest, abandoned leads, and the history of upgrade or additional lead implantation are more common in patients with significant venous obstruction expressed as the number of affected veins. With regard to the lead implant duration, all investigators agree that it has no influence on the risk of venous obstruction [10,15,16,20,22]. However, the findings of this study suggest that lead age may play an important role, as confirmed in the univariable regression analysis. The natural course of MLVSO remains unclear. An endothelial injury prompts an inflammatory response of the vessel wall with subsequent scarring [23]. Additionally, due to space constraints, the leads in the vein slow down the blood flow.

The data on the association between MLVSO and device infections are inconsistent. Some investigators underline the role of infection in venous obstruction [6,9,15,18], others disagree [1,4,5,7,8,10,13,14]. Our findings show that LRIE with or without pocket infection is more common in patients with a significant obstruction in multiple veins. These results might lead to the conclusion that infectious complications are more likely to occur in patients with MLVSO.

This present study shows that MLSVO is relatively less common in patients with a permanent AF. Previous reports on the impact of an AF and anticoagulation therapy on lead-related venous stenosis are contradictory [6,7,17,20]. One study [24] demonstrated that the incidence of significant venous stenosis at one site was lower in patients with an AF; however, the protective role of anticoagulation agents was not directly confirmed. It is possible that other lead-related factors (especially the number of leads) playing a role early in the development of venous stenosis outweigh the prothrombotic effects. 

The influence of MLVSO on the TLE difficulty, complexity, and effectiveness, and the occurrence of major complications has not been fully described. Only four reports addressed pre-extraction venous obstruction [5,9,17,18], and only two of them considered its influence on procedure complexity, providing contradictory findings [5,9]. Li X (202 patients) concluded that venous occlusion made the TLE more difficult [9], in contrast to Boczar K (133 patients) who stated that venous obstruction had no influence on the course of lead removal [5]. In this present study, the procedural difficulty was defined as a prolonged procedure or fluoroscopy time and the necessity of using advanced tools and techniques. However, much less has been written about unexpected obstacles or “technical problems” such as an obstructed entry site of implanted leads/subclavian region, polypropylene dilator collapse/fracture, lead-on-lead binding, a break in the target lead, the necessity of using an alternative vein access, the loss of broken lead fragments, or the dislodgement of functional leads. The occurrence of such unexpected technical problems requires the use of advanced tools and techniques. Once the problem has been detected, it has to be solved; however, it is not classified as a procedural complication. The risk of losing venous access significantly hinders lead replacement in the event of multilevel venous stenosis/occlusion and increases the complexity of the TLE procedure. Particularly when performing a TLE in patients with multilevel venous stenosis/occlusion, it is very important to introduce a vascular guidewire through the dilating sheath in place of the removed lead to maintain the possibility of introducing advanced tools in the event of problems with another lead removal or even a new lead implantation. In summary, patients with more extensive venous narrowing were likely to undergo longer and more complex procedures that required the use of additional tools. Major and minor complications, partial radiographic success, and the rate of TV damage were markedly higher in patients with multiple vein stenosis, whereas the rates of clinical and procedural success were lower in such individuals.

There was no association between the number of affected veins and mid- or long-term mortality (30-days, 1-year, 3-year-, and >3-year mortality).

### Study Limitations

This study has some limitations. Routine venography before the TLE was performed in all patients except those with contraindications (mainly renal failure). For this reason, this interesting subpopulation was excluded from this study. The database was integrated prospectively, but the analysis was performed retrospectively. In most patients, venography was performed on the side of lead insertion to avoid contrast overload. Well-developed collateral circulation in the neck permitted the evaluation of the contralateral brachiocephalic vein but not the subclavian and axillary veins in some patients.

## 5. Conclusions

The maximal narrowing measured at one point and the number of significantly affected veins are two forms of lead-related venous stenosis/occlusion. Multilevel lead-related venous stenosis/occlusion (MLVSO) better depicts the clinical consequences and procedural implications of venous involvement in the setting of the TLE, since multi-vessel disease is associated with significantly different effects. The underlying cause of multilevel lead-related venous stenosis or occlusion is probably lead-induced damage to the vascular wall; thus, the main risk factor for MLVSO is the number of implanted leads. MLVSO increases extraction complexity and major and minor complication rates and decreases the rates of clinical and procedural success but does not influence mortality after the TLE.

## Figures and Tables

**Table 1:** Comparison of patient-related risk factors for lead-related venous stenosis/occlusion in patients divided into groups according to the number of veins with significant venous obstruction.

Patient-Related Risk Factors for Lead-Related Venous Stenosis/Occlusion	Lack of Significant Narrowing (Grades 1, 2)	One Vein with Significant Narrowing (Grades 3, 4, and 5)	Two Veins with Significant Narrowing (Grades 3, 4, and 5)	Three, Four, or Five Veins with Significant Narrowing (Grades 3, 4, and 5)	Kruskal–Wallis ANOVA Test	Mann–Whitney U Test/Chi^2^ Test	All Examined Patients
Number of patients (group number)	1108 (1)	1152 (2)	665 (3)	77 (4)			3002
Values presented as	Mean ± SD N (%)	Mean ± SD N (%)	Mean ± SD N (%)	Mean ± SD N (%)	*p* 1, 2, 3, 4	*p* 1 vs. (2, 3, 4) 1 vs. (3, 4) 1 vs. 4	Mean ± SD N (%)
Patient’s age at first CIED implantation [years]	57.24 ± 15.79	58.45 ± 15.87	60.40 ± 15.14	55.46 ± 19.73	0.010	0.003 <0.001 0.837	58.43 ± 15.72
Patient’s age during TLE [years]	65.64 ± 66.92	66.92 ± 14.02	69.06 ± 13.33	67.09 ± 13.79	<0.001	0.009 <0.001 0.293	66.93 ± 14.11
Female	439 (39.62)	464 (40.28)	246 (36.99)	32 (41.56)	0.577		1181 (39.34)
UHD: ischemic heart disease	625 (56.41)	865 (57.73)	401 (60.30)	27 (48.05)	0.100		1728 (57.56)
UHD: primary cardiomyopathy	145 (13.09)	140 (12.15)	101 (15.19)	6 (7.729)	0.069	0.880 0.325 0.176	392 (13.06)
UHD: valvular heart disease	33 (2.978)	20 (1.736)	17 (2.556)	0 (0.00)	0.117		70 (2.332)
UHD: congenital, post-inflammatory, channelopathies, neurocardiogenic, and unknown	305 (27.53)	326 (28.30)	146 (21.96)	34 (44.15)	<0.001	0.874 0.527 <0.001	811 (27.02)
NYHA class [I-IV]	1.859 ± 0.687	1.796 ± 0.657	1.893 ± 0.656	1.662 ± 0.708	0.151		1.837 ± 0.970
NYHA III & IV (%)	170 (15.34)	142 (12.33)	106 (15.94)	11 (14.29)	0.206		429 (14.29)
LVEF average [%]	49.36 ± 15.60	49.07 ± 15.09	48.32 ± 14.95	50.29 ± 15.28	0.295		49.04 ± 15.25)
PASP [mm Hg]	30.34 ± 13.29	30.59 ± 31.35	31.45 ± 12.93	33.73 ± 14.09	0.127		30.77 ± 13.23)
RV diameter [mm]	31.59 ± 6.113	30.71 ± 31.04	31.05 ± 5.985	30.81 ± 5.575	<0.001	<0.001 0.002 0.199	31.11 ± 5.944)
Permanent atrial fibrillation	292 (26.35)	264 (22.92)	124 (18.65)	12 (15.58)	<0.001	<0.001 <0.001 0.031	692 (23.05)
Arterial hypertension	621 (56.05)	694 (60.24)	416 (62.56)	40 (51.94)	0.019	0.009 0.015 0.576	1771 (58.99)
Congestive heart failure	224 (20.22)	204 (17.71)	130 (19.55)	9 (11.68)	0.143		567 (18.89)
Diabetes (any)	221 (19.95)	240 (20.83)	140 (21.05)	10 (12.98)	0.217		611 (20.35)
Renal failure, mild	173 (15.61)	207 (17.96)	128 (19.25)	12 (15.58)	0.244		520 (17.32)
Renal failure, severe	73 (2.432)	27 (2.344)	13 (1.955)	4 (5.195)	0.137		73 (2.43)
Creatinine level [mg/dL]	1.144 ± 0.563	1.168 ± 0.737	1.164 ± 1.422	1.422 ± 1.357	0.286		1.165 ± 0.661
BMI [kg/m^2^]	28.27 ± 6.712	27.99 ± 4.412	27.90 ± 25.56	25.56 ± 3.818	<0.001	0.527 0.139 <0.001	28.61 ± 3.377
Valve implant	83 (7.491)	81 (6.446)	51 (7.669)	2 (2.600)	0.634		217 (7.229)
Mechanical valve	50 (4.510)	50 (4.340)	36 (5.414)	1 (1.299)	0.512		137 (4.560)
Previous sternotomy	169 (15.25)	178 (15.45)	89 (13.38)	11 (14.29)	0.754		447 (14.89)
Long-term anticoagulation	463 (41.79)	460 (39.93)	253 (38.05)	29 (37.66)	0.477		1205 (40.14)
Long-term antiplatelet treatment	487 (43.95)	515 (44.71)	309 (46.47)	36 (46.75)	0.730		1347 (44.87)
Charlson comorbidity index [points]	4.631 ± 3.693	4.780 ± 3.623	5.014 ± (3.570)	4.273 ± 2.259	0.027	0.043 0.017 0.464	4.767 ± 3.637

SD—standard deviation, N—number, CIED—cardiac implantable electronic device, TLE—transvenous lead extraction, UHD—underlying heart disease, NYHA—New York Heart Association functional class, LVEF—left ventricular ejection fraction, PASP—pulmonary artery systolic pressure, RV—right ventricle, and BMI—body mass index.

**Table 2:** Comparison of indication-related, system-related, and history of pacing-related risk factors for venous lead-related stenosis/occlusion in patients divided into groups according to the number of veins with significant venous obstruction.

Indication-, System- and History of Pacing-Related Risk Factors for Venous Stenosis/Occlusion	Lack of Significant Narrowing (Grades 1 and 2)	One Vein with Significant Narrowing (Grades 3, 4, and 5)	Two Veins with Significant Narrowing (Grades 3, 4, and 5)	Three, Four, or Five Veins with Significant Narrowing (Grades 3, 4, and 5)	Kruskal–Wallis ANOVA Test	Mann–Whitney U Test/Chi^2^ Test *p* 1 vs. (2, 3, 4)	All Examined Patients
Number of patients (group number)	1108 (1)	1152 (2)	665 (3)	77 (4)			3002
Values presented as	Mean ± SD N (%)	Mean ± SD N (%)	Mean ± SD N (%)	Mean ± SD N (%)	*p* 1,2,3,4	*p* 1 vs. (2, 3, 4) 1 vs. (3, 4) 1 vs. 4	Mean ± SD N (%)
TLE indication							
LRIE certain with or without pocket infection	136 (12.27)	165 (14.32)	129 (19.40)	20 (25.97)	<0.001	0.014 <0.001 <0.001	450 (14.99)
LRIE probable with or without pocket infection	51 (4.603)	69 (5.900)	47 (7.068)	7 (9.091)	0.074	0.029 0.012 0.064	174 (5.796)
Local/isolated pocket infection	97 (8.755)	83 (7.205)	64 (9.624)	4 (5.195)	0.259		248 (8.261)
All infections	284 (25.63)	317 (27.52)	240 (36.09)	31 (40.26)	<0.001	0.004 <0.001 0.006	872 (20.05)
Non-infectious indic. prophylactic	40 (3.610)	47 (4.080)	15 (2.256)	3 (3.896)	0.189		105 (3.498)
Non-infectious indic. therapeutic	784 (70.76)	788 (68.40)	410 (61.65)	43 (55.84)	<0.001	<0.001 <0.001 0.006	2025 (67.46)
Goal of TLE							
System removal—infection	282 (25.45)	317 (27.52)	239 (35.94)	31 (40.26)	<0.001	0.004 <0.001 0.007	869 (28.95)
Upgrade	150 (13.54)	134 (11.63)	82 (12.33)	6 (7.792)	0.314		372 (12.39)
Downgrade	40 (3.610)	55 (4.774)	42 (6.316)	2 (2.597)	0.094	0.032 0.029 0.820	139 (4.630)
Lead replacement	570 (51.44)	577 (50.09)	279 (41.96)	31 (40.26)	<0.001	0.017 <0.001 0.075	1457 (48.53)
Superfluous lead extraction	10 (0.903)	10 (0.869)	0 (0.00)	2 (2.597)	0.002	0.315 0.101 0.036	22 (0.733)
Redundant system removal	46 (4.152)	47 (4.080)	15 (2.256)	1 (1.299)	0.075	0.226 0.014 0.230	109 (3.631)
System removal—deferred reimplantation	10 (0.903)	12 (1.042)	8 (1.203)	4 (5.195)	0.006	0.352 0.154 <0.001	34 (1.133)
System and history of pacing							
Device type—PM (AAI, VVI, DDD, or CRT-P)	780 (70.94)	794 (68.92)	444 (66.77)	64 (83.12)	0.017	0.378 0.379 0.260	2082 (69.37)
Device type—ICD-V or ICD-D	263 (23.74)	268 (23.26)	146 (22.00)	10 (12.99)	0.211		687 (22.88)
Device type—CRT-D	26 (5.866)	90 (7.813)	75 (11.28)	3 (3.896)	<0.001	0.004 <0.00 0.510	233 (7.761)
Number of leads in the system before TLE	1.772 ± 0.679	1.834 ± 0.682	1.976 ± 1.249	1.857 ± 1.116	<0.001	<0.001 <0.001 0.064	1.825 ± 1.157
Presence of abandoned lead before TLE	84 (7.581)	93 (9.073)	104 (15.64)	28 (36.36)	<0.001	<0.001 <0.001 <0.001	309 (10.29)
Number of abandoned leads before TLE	0.095 ± 0.360	0.102 ± 0.36	0.205 ± 0.521	0.571 ± 0.856	<0.001	<0.001 <0.001 <0.001	0.134 ± 0.431
Multiple abandoned leads before TLE	20 (1.805)	22 (1.910)	103 (15.49)	13 (16.88)	<0.001	0.011 <0.001 <0.001	85 (2.831)
Number of leads in the heart before TLE	1.813 ± 0.686	1.930 ± 0.677	2.173 ± 0.799	2.403 ± 1.091	<0.001	<0.001 0.006 <0.001	1.953 ± 0.773
ICD leads before TLE	333 (30.05)	359 (31.16)	222 (33.83)	94 (18.18)	0.033	0.050 0.583 0.014	928 (30.81)
CS leads before TLE (for LA or LV pacing)	132 (11.91)	194 (16.84)	155 (23.31)	11 (14.29)	<0.001	<0.001 <0.001 0.679	492 (16.39)
Leads on the left side of the chest before TLE	1057 (95.40)	1098 (95.31)	634 (95.34)	55 (71.43)	<0.001	0.245 0.022 <0.001	2844 (94.74)
Leads on the right side of the chest before TLE	31 (2.798)	38 (3.299)	8 (1.203)	3 (3.896)	0.062	0.686 0.069 <0.539	80 (2.665)
Leads on both sides of the chest before TLE	20 (1.805)	16 (1.389)	22 (3.308)	19 (24.68)	<0.001	0.041 <0.001 <0.001	77 (2.665)
Previous TLE	46 (4.152)	44 (3.919)	41 (6.165)	4 (5.195)	0.104		135 (4.497)
Early CIED intervention	42 (3.794)	50 (4.340)	34 (5.113)	1 (1.299)	0.373		127 (4.232)
Upgrade or additional lead implantation	123 (11.10)	136 (11.81)	125 (18.80)	19 (24.68)	<0.001	0.004 <0.001 <0.001	403 (13.42)
Upgrade or downgrade with lead abandonment	39 (3.520)	63 (5.469)	69 (10.38)	16 (20.78)	<0.001	<0.001 <0.001 <0.001	187 (6.229)
Last CIED procedure before TLE excluding repair of unit pocket [months]	49.15 ± 37.67	49.10 ± 37.96	42.38 ± 33.09	53.63 ± 40.69	0.013	0.161 0.002 0.676	47.70 ± 36.97
Dwell time of oldest lead per patient before TLE [months]	101.7 ± 37.67	99.97 ± 75.45	103.5 ± 73.86	138.7 ± 97.89	0.006	0.772 0.120 <0.001	102.41 ± 75.95
Mean implant duration (per patient) before LTE [months]	95.35 ± 66.97	93.12 ± 67.24	93.81 ± 64.98	118.7 ± 74.84	0.038	0.618 0.753 0.011	94.75 ± 66.93
Cumulative lead dwell time before TLE [years]	14.65 ± 12.68	14.78 ± 11.74	16.95 ± 13.58	25.18 ± 22.34	<0.001	0.005 <0.001 <0.001	15.48 ± 13.00

SD—standard deviation, N—number, LRIE—lead-related infective endocarditis, TLE—transvenous lead extraction, PM—pacemaker, AAI—pacemaker with the lead tip in right atrium, VVI—pacemaker with the lead tip in right ventricle, DDD—dual chamber pacemaker, CRTP—cardiac resynchronization therapy pacemaker, ICD-V single chamber implantable cardioverter defibrillator, ICD-D—dual chamber implantable cardioverter defibrillator, CRTD—cardiac resynchronization therapy defibrillator, ICD—implantable cardioverter defibrillator (V or D), CS—coronary sinus, LA—left atrium, and CIED—cardiac electronic implantable device.

**Table 3:** Factors influencing multilevel lead-related venous stenosis/occlusion. Results of univariate and multivariable regression analysis.

	Any Level of LRVSO					Two-Level Nature of LRVSO					Three- or More Level Nature of LRVSO				
	Univariable Regression		Multivariable Regression			Univariable Regression		Multivariable Regression			Univariable Regression		Multivariable Regression		
	OR	*p*	OR	95%CI	*p*	OR	*p*	OR	95%CI	*p*	OR	*p*	OR	95%CI	*p*
Patient’s age during first system implantation [years]	1.007	0.003	1.005	0.999 −1.012	0.115	1.011	<0.001	1.015	1.007 −1.024	<0.001	1.005	0.580			
Patient’s age during TLE [years]	1.009	0.002				1.016	<0.001				0.993	0.352			
Baseline heart disease: post-inflammatory, congenital, channelopathies, neurocardiogenic, or unknown	1.015	0.768				0.929	0.527				2.634	<0.001	1.358	0.735 −2.507	0.328
RV diameter	0.977	<0.001	0.980	0.966 −0.993	0.004	0.980	0.018	0.991	0.972 −1.010	0.344	0.971	0.183			
AF permanent	0.749	0.001	0.702	0.570 −0.865	0.001	0.620	<0.001	0.548	0.415 −0.723	<0.001	0.493	0.034	0.537	0.251 −1.152	0.110
Arterial hypertension	1.224	0.009	1.121	0.936 −1.342	0.213	1.267	0.015	1.165	0.924 −1.468	0.196	0.875	0.574			
Body mass index	0.988	0.146				0.977	0.033	0.972	0.949 −0.995	0.019	0.871	<0.001	0.864	0.808 −0.925	<0.001
Charlson’s index	1.013	0.210				1.020	0.136				0.958	0.227			
Device type—CRT-D	1.543	0.004	0.762	0.499 −1.163	0.208	1.861	<0.001	0.695	0.418 −1.154	0.159	0.672	0.512			
Number of leads in the system before TLE	1.518	<0.001				1.890	<0.001				1.409	0.071			
Presence of abandoned lead before TLE	1.661	<0.001				2.706	<0.001				7.139	<0.001			
Number of abandoned leads before TLE	1.459	<0.001				2.058	<0.001				3.642	<0.001			
Multiple abandoned leads before TLE	1.916	0.012				3.314	<0.001				10.46	<0.001			
Number of leads in the heart before TLE	1.532	<0.001	1.332	1.111 −1.596	0.002	2.004	<0.001	1.706	1.366 −2.132	<0.001	2.531	<0.001	1.688	1.081 −2.635	0.021
≥ 4 leads before TLE	2.692	0.001				5.232	2.910				14.01	<0.001			
≥ 5 leads before TLE	10.69	<0.001				10.69	0.027				46.48	<0.001			
ICD leads—before TLE	0.991	0.172				0.990	0.292				0.467	0.018	0.543	0.256 −1.153	0.111
CS lead presence before TLE (for LA or LV pacing)	1.341	<0.001	1.247	1.047 −1.485	0.013	1.491	<0.001	1.274	1.028 −1.578	0.027	1.170	0.391			
Leads both side of the chest before TLE	1.700	0.043	1.015	0.520 −1.981	0.965	3.230	<0.001	1.513	0.791 −2.896	0.211	18.67	<0.001	7.203	2.716 −19.10	<0.001
Upgrading or additional lead implantation	1.395	0.004	0.815	0.591 −1.125	0.213	1.964	<0.001	0.917	0.628 −1.339	0.653	2.747	<0.001	0.648	0.188 −2.237	0.492
Upgrading or downgrading with lead abandonment	2.373	<0.001	2.488	1.483 −4.174	0.001	3.705	<0.001	2.286	1.269 −4.119	0.006	7.731	<0.001	2.789	0.618 −12.59	0.182
Dwell time of the oldest lead before TLE	1.003	0.593				1.013	0.099				1.061	<0.001			
Cumulative dwell time of leads (in years) before TLE	1.007	0.016	0.998	0.990 −1.007	0.714	1.016	<0.001	0.996	0.986 −1.005	0.345	1.036	<0.001	0.992	0.973 −1.011	0.416
Strong connective tissue scar connection of the lead with heart structures (any)	0.744	0.014				0.649	0.873				0.708	0.400			
Strong connective tissue scar connection of the lead with RA wall	0.568	0.006	0.603	0.395 −0.920	0.019	0.669	0.125				0.880	0.835			
TV regurgitation [grades I and II]	1.066	0.502				0.916	0.475				0.548	0.025	0.583	0.301 −1.127	0.108
Impact of the multilevel nature of lead-related venous stenosis/occlusion on TLE indications. Results of univariable regression analysis															
	Any level of LRVSO					Two-level nature of LRVSO					Three- or more level nature of LRVSO				
	OR	95%CI			*p*	OR	95%CI			*p*	OR	95%CI			*p*
LRIE certain with or without pocket infection	1.422	1.145–1.766			<0.001	1.798	1.393–2.321			<0.001	2.451	1.411–4.258			<0.001
LRIE probable with or without pocket infection	1.450	1.037–2.029			0.030	1.652	1.113–2.452			0.013	2.151	0.939–4.926			0.070
All infections	1.307	1.107–1.545			0.002	1.670	1.364–2.045			<0.001	1.961	1.209–3.182			0.006
Sure vegetation	1.454	1.162–1.819			<0.001	1.982	1.525–2.576			<0.001	3.019	1.740–5.238			<0.001
Downgrading (TLE ind.)	1.541	1.035–2.294			0.033	1.680	1.051–2.636			0.030	0.846	0.199–3.593			0.821
Lead replacement (TLE ind.)	0.833	0.718–0.986			0.017	0.690	0.571–0.833			<0.001	0.601	0.372–0.971			0.037
Superfluous lead extraction (TLE ind.)	3.031	0.651–14.11			0.157	0.300	0.066–1.379			0.121	3.031	0.651–14.11			0.157
System removal—reimplantation differed (TLE ind.)	6.234	1.905–20.40			0.002	0.483	0.267–0.872			0.016	0.314	0.043–2.316			0.255

RV—right ventricle, AF—atrial fibrillation, CRTD—cardiac resynchronization therapy defibrillator, TLE—transvenous lead extraction, ICD—implantable cardioverter defibrillator, CS—coronary sinus, LA—left atrium, LV—left ventricle, RA—right atrium, TV—tricuspid valve, LRIE—lead-related infective endocarditis, and TLE—transvenous lead extraction.

**Table 4:** TLE efficacy and complications and long-term mortality after TLE in patients divided into groups according to the number of veins with significant venous obstruction.

TLE Efficacy and Complications and Long-Term Mortality after TLE	Lack of Significant Narrowing (Only 1 and 2 Degrees)	One Vein Involved with Significant Narrowing (3, 4, and 5 Degrees)	Two Veins Involved with Significant Narrowing (3, 4, and 5 Degrees)	Three, Four or Five Veins Involved with Significant Narrowing (3, 4, and 5 Degrees)	Kruskal–Wallis ANOVA, *	Chi^2^ Test	All Examined Patients
Number of patients (number of the group)	1108 (1)	1152 (2)	665 (3)	77 (4)			3002
Presented values	N (%)	N (%)	N (%)	N (%)	*p* 1,2,3,4	*p* 1 vs. (2, 3, 4) 1 vs. (3, 4) 1 vs. 4	N (%)
TLE efficacy and complications							
Major Complications (any)	19 (1715)	27 (2.355)	11 (1.645)	7 (9.091)	0.002	0.336 0.410 <0.001	64 (2.132)
Hemopericardium	5 (0.542)	18 (1.563)	7 (1.053)	4 (5.195)	0.003	0.336 0.213 <0.001	41 (1.366)
Hemothorax	1 (0.090)	1 (0.087)	2 (0.301)	1 (1.299)	0.006	0.189 0.094 <0.001	5 (0.167)
Tricuspid valve damage during TLE	5 (0.542)	8 (0.694)	2 (0.301)	1 (1.299)	0.399		17 (0.566)
Rescue cardiac surgery	7 (0.632)	17 (1.476)	6 (0.902)	5 (6.493)	<0.001	0.021 0.036 <0.001	35 (1.166)
Minor complications (any)	52 (5.866)	81 (7.031)	55 (8.271)	9 (11.69)	0.035	0.040 0.013 0.011	210 (6.995)
Death procedure-related (intra- and post-procedural)	2 (0.181)	3 (0.260)	1 (0.150)	0 (0.00)	0.985		6 (0.200)
Death indication-related (intra- and post-procedural	1 (0.090)	1 (0.087)	0 (0.00)	0 (0.00)	0.721		2 (0.067)
Partial radiological success (remained tip or <4 cm lead fragment)	34 (3.069)	40 (3.472)	28 (4.211)	8 (10.39)	0.002	0.168 0.049 <0.001	110 (3.664)
Full Clinical Success	1090 (98.38)	1128 (97.92)	654 (98.36)	72 (93.51)	0.029	0.381 0.356 0.003	2944 (98.07)
Full Procedural Success	1065 (96.12)	1100 (95.85)	634 (95.34)	68 (88.31)	0.012	0.199 0.109 <0.001	2867 (95.50)
TLE-related TV dysfunction (noncomplete data)							
TR remained unchanged	828/895 (92.51)	870/947 (91.87)	478/544 (87.87)	54/61 (88.52)	0,168		2230/2447 (91.13)
Increase in TR of 1 degree	49/895 (5.475)	55/947 (5.808)	49/544 (9.007)	5/61 (8.197)	0.071	0.201 0.019 0.516	158/2447 (6.457)
Increase in TR of 2 degrees	14/895 (1.564)	19/947 (2.006)	14/544 (2,574)	2/61 (3.279)	0.584		49/2447 (2.002)
Increase in TR of 3 degrees	4/895 (0.447)	3/947 (3.168)	3/544 (5.515)	0/61 (0.00)	0.606		10/2447 (0.409)
Increase in TR of 2 degrees and up to 4 degrees	6/1106 (0.596)	8/1150 (0.696)	3/663 (4.525)	0/77 (0,00)	0.822		17/2996 (0.567)
Damage of horde tendinea during TLE	29/988 (2.935)	29/1027 (2.824)	34/598 (5.686)	5/58 (8.621)	0.009	0.141 0.004 0.158	97/2681 (3.618)
Short-, mid-, and long-term mortality after TLE							
First two days mortality (first 48 h)	4 (0.361)	5 (0.434)	1 (0.150)	0 (0.00)	0.437 *		10 (0.333)
1-month mortality after TLE; 2–30 days *n* (% of patients with follow-up longer than 2 days)	13 (1.178)	10 (0.872)	6 (0.904)	1 (1.299)	0.437 *		30 (0.003)
1-year mortality after TLE (31–365 days); *n* (% of patients with follow-up longer than 30 days)	71 (6.717)	67 (6.004)	42 (6.573)	10 (13.70)	0.437 *		190 (6.767)
3-year mortality after TLE (366–1095 days); *n* (% of patients with follow-up longer than 365 days	75 (8.126)	117 (11.74)	64 (11.66)	3 (4.918)	0.437 *		259 (10.49)
Death late >3 years after TLE (after 1095 days); *n* (% of patients with follow-up longer than 1095 days)	147 (22.62)	168 (22.80)	117 (29.40)	12 (25.53)	0.437 *		444 (24.87)

* Log rank test. N—number, TLE—transvenous lead extraction, and TR—tricuspid valve regurgitation.

## Data Availability

All data generated or analyzed during this study are included in this published article.

## Supplementary Materials

The following supporting information can be downloaded at: https://www.mdpi.com/article/10.3390/medicina60020336/s1, Table S1: The rate of different degree of maximal VSO in compared groups with different length of narrowing/occlusion; Table S2: TLE procedure complexity in patients divided into groups according to the number of lead related significantly stenosed/occluded vein; Figure S1: Univariate regression analysis of the influence of multilevel venous obstruction/stenosis on the difficulty of lead extraction, Figure S2: Kaplan Meier survival curves of patients with MLVSO undergoing TLE.
