# Supplementary material for: Multilevel Venous Obstruction in Patients with Cardiac Implantable Electronic Devices
Source: Medicina (Kaunas). 2024 Feb 17;60(2):336. doi: 10.3390/medicina60020336 (PMC10890105; doi:10.3390/medicina60020336)
Supplement: Supplementary file 1 [file medicina-60-00336-s001.zip › medicina-2802797-supplementary.pdf]

**Multilevel venous obstruction in patients with cardiac implantable electronic devices**

## Supplementary materials

Table S1 presents the relationship between the rate of different MLVSO severity (maximal narrowing) in groups with different narrowing/occlusion extent expressed as a few significantly (3-5th degree) involved veins. The rate of different narrowing/occlusion extent in compared groups with different degrees of maximal MLVSO is also presented. The lower part of the Table 3 presents mirror analysis of different narrowing / occlusion length rate in compared groups with different degree of maximal MLVSO (another calculation of percentages). The results seem to confirm a relationship between single maximal narrowing appearance and a number of significantly problematic veins (extent of narrowing).

Table S1 The rate of different degree of maximal VSO in compared groups with different length of narrowing / occlusion (A) and the rate of different length of narrowing / occlusion in compared groups with different degree of maximal VSO (B).

| **A. Rate of different degree of maximal VSO in compared groups with different length of narrowing / occlusion** | **Lack of significant narrowing (only 1,2 degree)** | **One vein involved with significant narrowing (3,4,5 degree)** | **Two veins involved with significant narrowing (3,4,5 degree)** | **Three, four or five veins involved with significant narrowing (3,4,5 degree)** | **All examined patients** |
|---|---|---|---|---|---|
| Number of patients number of the group | **1108** | **1152** | **665** | **77** | **3002** |
| Presented values | Number (%) | Number (%) | Number (%) | Number (%) | Number (%) |
| Lack of narrowing (1 degree) | 510 (46,03) | 0 (0,00) | 0 (0,00) | 0 (0,00) | 510 (16,99) |
| Small/ mild narrowing (2 degree) | 598 (53,97) | 0 (0,00) | 0 (0,00) | 0 (0,00) | 598 (19,92) |
| Moderate narrowing (3 degree) | 0 | 543 (47,13) | 76 (11,43) | 2 (2,597) | 621 (20,69) |
| Severe narrowing (4 degree) | 0 | 407 (35,33) | 185 (27,82) | 6 (7,792) | 598 (19,990 |
| Total occlusion (5 degree) | 0 | 202 (17,53) | 404 (60,75) | 69 (89,61) | 675 (22,49) |
| All examined patients | 1108 (100,0) | 1152 (100,0) | 665 (100,0) | 77 (100,0) | 3002 (100,0) |
| | | | | | |
| **B. Rate of different length of narrowing / occlusion in compared groups with different degree of maximal VSO** | **Lack of significant narrowing (only 1,2 degree)** | **One vein involved with significant narrowing (3,4,5 degree)** | **Two veins involved with significant narrowing (3,4,5 degree)** | **Three, four or five veins involved with significant narrowing (3,4,5 degree)** | **All examined patients** |
| Number of patients number of the group | **1108** | **1152** | **665** | **77** | **3002** |
| Presented values | Number (%) | Number (%) | Number (%) | Number (%) | Number (%) |
| Lack of narrowing (1 degree) | 510 (100,0) | 0 (0,00) | 0 (0,00) | 0 (0,00) | 510 (100,0) |
| Small narrowing (2 degree) | 598 (100,0) | 0 (0,00) | 0 (0,00) | 0 (0,00) | 598 (100,0) |
| Moderate narrowing (3 degree) | 0 (0,00) | 543 (87,44) | 76 (12,24) | 2 (0,322) | 621 (100,0) |
| Severe narrowing (4 degree) | 0 (0,00) | 407 (68,06) | 185 (30,94) | 6 (1,003) | 598 (100,0) |
| Total occlusion (5 degree) | 0 (0,00) | 202 (29,93) | 404 (59,85) | 69 (10,22) | 675 (100,0) |
| All examined patients | 1108 (36,91) | 1152 (38,37) | 665 (22,15) | 77 (2,565) | 3002 (100,0) |

A comparative analysis of the difficulty of lead extraction depending on MLVSO showed that time of the procedure, the necessity of non-functional lead abandonment, the necessity of non-functional superfluous lead extraction, the appearance of any technical problem during TLE (such as a block in lead venous entry, lead to lead strong scar connection, Byrd dilator collapse/torsion / "fracture", extracted lead collapse/rupture during extraction) and the number of technical problem were significantly more frequent, especially in groups of patients with two or more problematic veins with significant stenosis/occlusion. There were positive correlations between number of levels of MLVSO and number of big technical problems (Spearman r coefficient = 0,069; p<0,001) and procedure duration time (Spearman r coefficient = 0,108; p<0,001). Similarly, the need for additional tools utility, such as mechanically driven sheaths, metal sheath, lasso catheter/snare or basket catheter, was much more frequent in this group of patients (Table S2)

## Table S2

Table S2 TLE procedure complexity in patients divided into groups according to the number of lead related significantly stenosed / occluded vein

| | **Lack of significant narrowing (only 1,2 degree)** | **One vein involved with significant narrowing (3,4,5 degree)** | **Two veins involved with significant narrowing (3,4,5 degree)** | **Three, four or five veins involved with significant narrowing (3,4,5 degree)** | **Kruskal-Wallis ANOVA Test** | **“U” Mann-Whitney / Chi$^2$ Tests** | **All examined patients** |
|---|---|---|---|---|---|---|---|
| Number of patients ( number of the group) | **1108<br>(1)** | **1152<br>(2)** | **665<br>(3)** | **77<br>(4)** | | | **3002** |
| Presented values | Mean ± sd<br>N (%) | Mean ± sd<br>N (%) | Mean ± sd<br>N (%) | Mean ± sd<br>N (%) | **P<br>1,2,3,4** | **P<br>1 *vs* (2,3,4)<br>1 *vs* (3,4)<br>1 *vs* 4** | Mean ± sd<br>N (%) |
| **TLE procedure complicity** | | | | | | | |
| Procedure duration (skin to skin) [minutes] | 58,26 ±24,17 | 58,18 ± 20,94 | 62,87± 31,57 | 81,30 ± 50,28 | <0,001 | 0,100<br><0,001<br><0,001 | 59,84 ± 26,15 |
| Procedure duration (sheath to sheath) [minutes] | 12,81 ± 20,34 | 12,89 ± 17,26 | 18,59 ± 29,04 | 38,81 ± 53,57 | <0,001 | <0,001<br><0,001<br><0,001 | 14,78 ± 23,39 |
| Average time of single lead extraction. (sheath-to sheath / number of extracted leads) [minutes] | 8,259 ± 13,23 | 8,055 ± 10,23 | 9,464 ± 13,91 | 16,23 ± 18,15 | <0,001 | 0,264<br><0,001<br><0,001 | 8,649 ± 12,55 |
| All leads were extracted | 860 (77,61) | 855 (74,22) | 500 (75,19) | 61 (79,21) | 0,229 | | 2276 (75,82) |
| Functional lead was left for continuous use | 241 (21,75) | 291 (25,26) | 162 (24,36) | 13 (16,88) | 0,087 | 0,074<br>0,298<br>0,251 | 707 (23,55) |
| Non-functional lead was left | 4 (0,361) | 2 (0,174) | 2 (0,301) | 4 (5,195) | 0,093 | 0,961<br>0,332<br><0,001 | 12 (0,400) |
| Non-functional, superfluous lead was extracted | 81 (7,310) | 87 (7,552) | 99 (14,89) | 22 (28,71) | <0,001 | <0,001<br><0,001<br><0,001 | 289 (9,627) |
| Technical problem during TLE (any) | 204 (18,41) | 213 (18,49) | 162 (24,36) | 29 (37,66) | <0,001 | 0,087<br><0,001 | 608 (20,25) |

| | | | | | | | |
|---|---|---|---|---|---|---|---|
| | | | | | | <0,001 | |
| Block in lead venous entry (subclavian region) | 79 (7,130) | 80 (6,944) | 70 (10,53) | 12 (15,58) | <0,001 | 0,128<br>0,002<br>0,004 | 241 (8,028) |
| Lead to lead strong connection with scar | 62 (5,596) | 74 (6,424) | 60 (9,023) | 16 (20,78) | <0,001 | 0,017<br>0,765<br>0,138 | 212 (7,062) |
| Byrd dilator collapse / torsion / "fracture" | 35 (3,159) | 32 (2,778) | 29 (4,361) | 7 (9,091) | 0,038 | 0,663<br>0,100<br>0,025 | 103 (3,341) |
| Extracted lead break / rupture during extraction | 47 (4,242) | 64 (5,556) | 39 (5,865) | 12 (15,58) | <0,001 | 0,022<br>0,182<br><0,001 | 162 (5,396) |
| Necessity to change venous approach | 25 (2,256) | 34 (2,951) | 26 (3,910) | 16 (20,78) | <0,001 | 0,011<br><0,001<br><0,001 | 101 (3,364) |
| Functional lead dislodgement | 14 (1,264) | 13 (1,128) | 3 (0,451) | 0 (0,00) | 0,298 | | 30 (0,999) |
| Loss of free lead fragment | 4 (0,361) | 2 (0,174) | 3 (0,451) | 2 (2,597) | 0,007 | 0,961<br>0,332<br>0,006 | 11 (0,366) |
| Number of big technical problems | 1,321 ±0,668 | 1,357 ± 0,668 | 1,379 ± 0,632 | 1,731 ± 1,151 | <0,001 | 0,091<br><0,001<br><0,001 | 1,368 ± 0,696 |
| One technical problem only | 126 (11,37) | 123 (10,68) | 95 (14,29) | 15 (19,48) | <0,001 | 0,648<br>0,055<br>0,035 | 359 (11,96) |
| Two technical problems | 30 (2,708) | 33 (2,865) | 31 (4,662) | 6 (7,792) | <0,001 | 0,103<br>0,026<br><0,001 | 100 (3,331) |
| Three or more technical problems | 9 (0,812) | 12 (1,042) | 9 (1,353) | 5 (6,494) | <0,001 | | 35 (1,155) |
| **Utility of additional tools** | | | | | | | |
| Evolution (old and new) or TighRail | 12 (1,063) | 13 (1,128) | 15 (2,259) | 6 (7,792) | <0,001 | 0,198<br>0,005<br><0,001 | 46 (1,533) |
| Metal sheath | 72 (6,498) | 80 (6,944) | 67 (10,09) | 12 (15,58) | <0,001 | 0,043<br><0,001<br><0,001 | 231 (7,697) |
| Lasso catheter / snare | 29 (2,617) | 40 (3,472) | 24 (3,614) | 8 (10,39) | <0,001 | 0,103<br>0,029<br><0,001 | 101 (3,366) |
| Basket catheter | 13 (1,173) | 5 (0,434) | 7 (1,054) | 2 (2,597) | 0,047 | 0,121<br>0,877<br>0,259 | 27 (0,900) |

sd – standard deviation, N – number, TLE – transvenous lead extraction

TLE procedure is much more time-consuming, difficult and skill-demanding in patients with two or more veins involved with significant narrowing. The OR of influence of the number of levels of MLVSO and above mentioned complications is also presented in Figure S1.

**Figure S1**

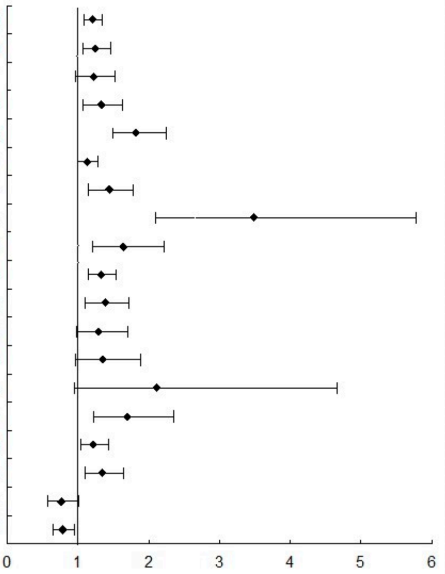


| | Univariable regression results | | |
|---|---|---|---|
| | OR | 95%CI | P |
| Technical problem during TLE (any) | 1.250 | 1.125-1.389 | <0.001 |
| Block in lead venous entry (subclavian region) | 1.296 | 1.118-1.503 | <0.001 |
| Byrd dilator collapse / torsion / "fracture" | 1.248 | 1.000-1.558 | 0.050 |
| Extracted lead break / rupture during extraction | 1.354 | 1.102-1.664 | 0.004 |
| Necessity to change venous approach | 1.857 | 1.513-2.279 | <0.001 |
| One technical Problem only | 1.154 | 1.016-1.310 | 0.027 |
| Two Technical Problems | 1.460 | 1.174-1.814 | <0.001 |
| Three or more Technical Problems | 3.503 | 2.119-5.791 | <0.001 |
| Utility of Evolution (old and new) or TighRail | 1.667 | 1.239-2.244 | <0.001 |
| Utility of metal sheath | 1.341 | 1.154-1.558 | <0.001 |
| Utility of Lasso catheter / snare | 1.389 | 1.115-1.731 | 0.003 |
| Major Complications (any) | 1.309 | 0.996-1.720 | 0.053 |
| Hemopericardium | 1.362 | 0.977-1.899 | 0.068 |
| Haemothorax | 2.115 | 0.955-4.683 | 0.065 |
| Rescue cardiac surgery | 1.706 | 1.230-2.367 | <0.001 |
| Minor complications (any) | 1.221 | 1.045-1.428 | 0.012 |
| Partial radiological success | 1.342 | 1.098-1.641 | 0.004 |
| Full Clinical Success | 0.759 | 0.574-1.003 | 0.052 |
| Full Procedural Success | 0.787 | 0.650-0.935 | 0.014 |

Figure S1 Univariate regression analysis of the influence of multilevel venous obstruction/stenosis on the difficulty of lead extraction

The log-rank analysis of the Kaplan Meier survival curves showed no effect of the multi-level nature of venous stenosis/occlusion on long-term survival (log rank p = 0.437, Figure S2).

**Figure S2**

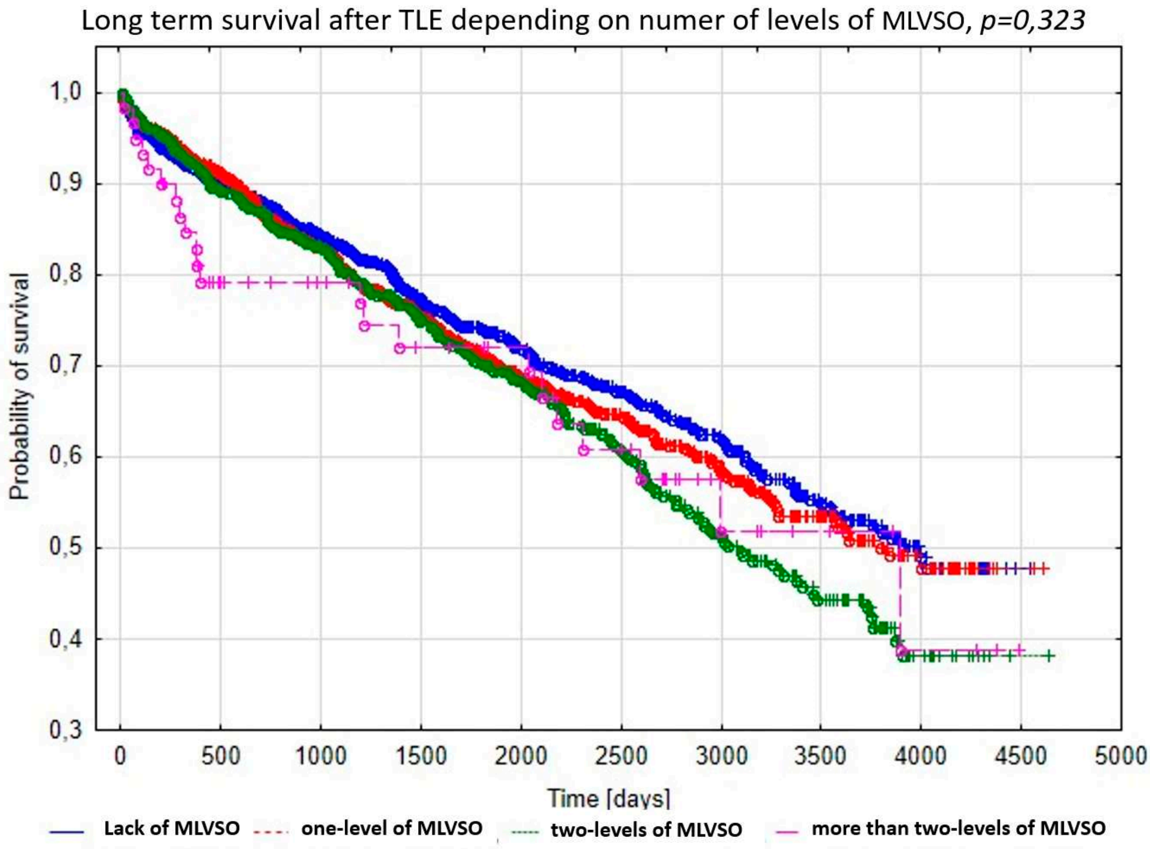


**Figure S2** Kaplan Meier survival curves of patients with MLVSO undergoing TLE
